# A hybrid mode semi circular shaped frequency reconfigurable antenna for multiband communication

**DOI:** 10.1038/s41598-025-18151-2

**Published:** 2025-10-03

**Authors:** Kanniyappan Vinayagam, Rajesh Natarajan

**Affiliations:** School of Electronics Engineering, Vellore Institute of Technology, Vellore, 632014 Tamil Nadu India

**Keywords:** Hybrid antenna, Reconfigurable antenna, PIN diode, Axial ratio, Ultra-wideband, Multiband communication, Electrical and electronic engineering, Applied physics, Engineering

## Abstract

This paper presents a hybrid mode semi-circular-shaped frequency reconfigurable antenna for ultra-wideband (UWB) and multiband communication. The proposed antenna is designed on a FR-4 substrate with a thickness of 1.6 mm, a relative permittivity of 4.2, and a compact size of 40 mm x 30 mm. Re-configurability is enabled by four PIN diodes, allowing the antenna to switch between UWB and various modes. The antenna operates in five different modes. In Mode 1 (all diodes OFF), the antenna operates as a UWB antenna covering  3.89 – 12.94 GHz with a peakgain of 6.93 dBi.It exhibits an axial ratio (AR) below 3 dB at 10.7 GHz, achieving a circular polarization bandwidth (AR BW) of 330 MHz from (10.53–10.86) GHz. Activating specific PIN diode combinations allows the antenna to function in different multiband configurations, supporting frequencies for 5G sub-6 GHz, WiMAX, and radar applications. This antenna’s versatility, through its frequency re-configurability, makes it an ideal candidate for modern communication systems.

## Introduction

Reconfigurable antennas are capable of instantly altering their Radiation pattern, polarization, and frequency^1^. Due to the developments in wireless communication systems, important specifications must be met when building antennas for multiband and ultra-wideband (UWB) operation. UWB antennas have a wide frequency range; they can transmit data at high speeds and provide accurate localization. This brief-range wireless communication has received significant interest from researchers worldwide. UWB technology offers high-speed communication with lower consumption of electricity than current narrowband approaches. UWB systems have many different applications including imaging for healthcare, ground-penetrating radar systems, defense-related tracking devices, and individual high-speed communication networks. Multiband antennas are designed to operate on many discrete frequency bands. It is compatible with a variety of wireless technologies, including GSM, LTE, Wi-Fi, and Bluetooth…etc.

In^2^, CPW fed monopole antenna with lumped RLC components act as switches to achieve frequency reconfiguration in order to cover six different frequency bands including WLAN, Wi-Fi, WiMAX, and UMTS. A multiband antenna using inverted U and E shaped stubs for digital broadcasting, medical telemetry, WLAN, WiMAX, fixed satellite communication and sub 6 GHz 5G applications is proposed in ^3^. A monopole antenna printed on single element and CPW fed with a pair of split ring resonators and PIN diodes is used ultra-wideband communication^4^. Re-configurability is introduced by connecting several stubs to the monopole feed line using GaAs FET switches, as defined in a symmetrical reconfigurable UWB circular disk monopole antenna for cognitive radio application^5^. Monopole antenna is designed to a single port and single substrate for reconfiguration function, to a combination of PIN diodes for mode of operation switching and Varactor diodes for continuously tuning the frequency of operation throughout the available Wideband and Narrow band, has been shown to be effective for a Reconfigurable antenna in CR applications^6^. In^7^ this work introduces a frequency-reconfigurable Filtenna capable of tuning between UWB and C-band states for cognitive system applications. Multifunctional antenna is two switchable notches for UWB operation and can be reconfigured for dual-band WiMAX and WLAN applications. So, finally seven states could be achieved from a single antenna using an electronic switching^8^. By adjusting the states of four diodes, the proposed antenna, which combines the monopole and a tapered slot, can be configured to produce two different types of radiation patterns^9^. Re configurability is enabled using PIN diodes. So, it is allowing a single antenna to combine UWB and reconfigurable Narrow band capabilities^10^. A reconfigurable feeding network is offered as a new design concept to implement the frequency switching operation of the ultra-wideband (UWB) state and dual wireless local area network states. The connected feed network consists of a flexible broadband filtering mechanism, and three-pole helical bands filtering process. The feed network utilizes multiple RF directions by controlling DC biased p-i-n diodes so realizing the frequency shifting function^11^. In^12^ D shaped monopole antenna with wide dual band dual sense circular polarization is proposed. The ICS and TIS structures achieving a wide 3 dB axial ratio bandwidth in both bands as compared to two variations of the design. In a proposed semi-circular UWB antenna, the ground plane will be modified in parallel to improve isolation and reduce the coupling current^13^. To develop a frequency reconfigurable antenna for cognitive radio applications are proposed using shape memory alloy^14^. The dual mode reconfigurable antenna achieves circularly polarized properties by inserting a gap in the annular ring^15^, for wideband wireless communication applications. UWB antenna is designed to achieve dual band notched frequencies by using a DMS and slot etched in the radiation patch^16^. In^17^ order to obtain dual bands at higher and lower frequencies, an imperfect ground structure is etched into the ground plane which is used to create reconfigurable EBG . This technique reduces the height of a crossed Magneto Electric Dipole antenna by up to 50% while retaining an ultra-wideband operation bandwidth of 93 to 360 MHz^18^.  A monopole antenna covers the UWB frequency spectrum range using different controlling capability for both WLAN and X-band communications^19^. The design and analysis of a small UWB MIMO antenna with band rejection and isolation enhancement structures influenced by fractals are introduced in^20^-^21^. UWB antennas exposed increasing interest in compact designs for MIMO application^22 which used ^. octagonal radiators and parasitic stubs In^23^, an elliptically slotted antenna achieved dual-band rejection using S-shaped and C-shaped structures for WLAN and X-band suppression. A compact fractal antenna with semi-circular DGS was proposed in^24^, offering dual high-frequency band notches with 96% efficiency and stable group delay. Further, a tri-band notched UWB antenna was presented in^25^, effectively eliminating C, WLAN, and X-band interferences through structural modifications. A quad-band notched design using slots, SSRR, and via holes is demonstrated in^26^, extending UWB performance with precise band control. These works highlight various techniques such as fractal structures, parasitic slits, and DGS for achieving wide bandwidth, improved isolation, and targeted band rejection closely aligning with the objectives of the present work. In this paper the proposed semi-circle UWB antenna provides a versatile solution for various communication and radar applications. Its reconfigurability, combined with good performance metrics, makes it a robust choice for scenarios requiring different frequency bands and operational modes. Experimental validation supports the effectiveness of the design, demonstrating its practical feasibility for real-world applications.

## Design procedure for the proposed antenna

### Antenna design

**Fig. 1:**
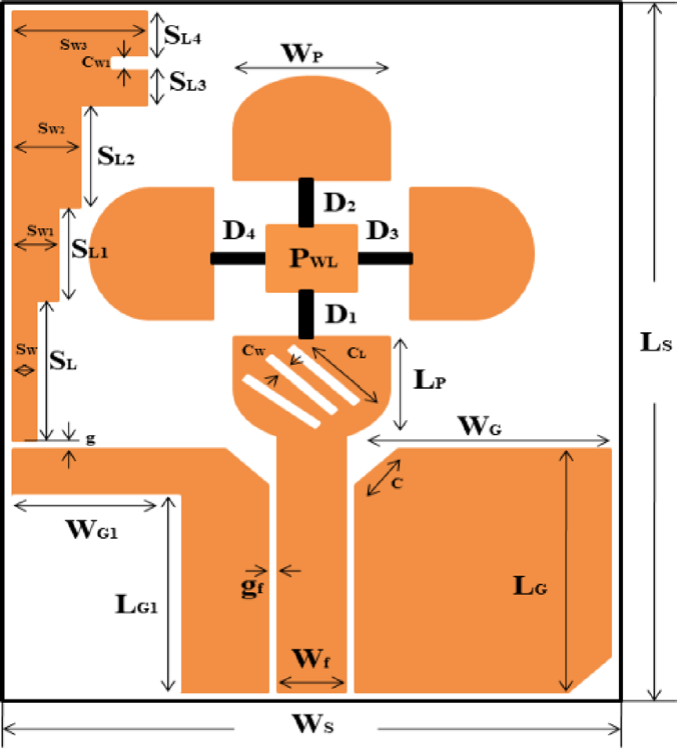
Geometric representation of the presented reconfigurable UWB antenna.

Figure.1 describes the geometry of the proposed UWB antenna. Figure 1 shows a circular patch antenna with a co-planner waveguide (CPW) feed. The antenna size is 40 mm × 30 mm printed on FR4 substrate (Ls×Ws) with a dielectric constant ε_r_ = (4.4), and thickness h = 1.6 mm . The antenna geometry is matched to 50 ohm CPW asymmetric feed line whose length and width is L = 17.08 mm and W_f_=3 mm respectively. Two equal spaces of width g = 0.5 mm are maintained between the ground planes (width W_G_=13, L_G_=16.5and length W_G1_=6, L_G1_=14) and the center feed strip. The complete geometry of the proposed antenna is shown in Table 1. The designed antenna is simulated and optimized using the CST Simulation Studio.

The initial design for UWB radiator patch length (L_p_) is calculated as (1–2),

1 $$L_{p} = {\text{ }}\lambda _{g} /4 = \frac{c}{{~4fr\sqrt {~\varepsilon eff} }}$$

2 $${\rm E}_{{eff}} = \frac{{\varepsilon r + 1}}{2}$$

λ_g_- Guided wavelength,

Ε_eff_- Effective dielectric constant,

c - Velocity of light,

f_r_- Resonance frequency.

**Table 1:** Dimensions of proposed antenna.

Variables	Units (mm)	Variables	Units (mm)
Ls	40	S_W2_	3.5
W_G1_	6	P_WL_	4
S_L1_	5	W_G_	13
S_W_	1	G	0.1
L_P_	6	S_L4_	2.4
Ws	30	S_W3_	6
g_f_	0.5	C_W_	0.4
S_L2_	5	L_G1_	14
S_W1_	1	S_L_	8.4
W_P_	6.93	C	1.41
L_G_	16.5	C_W1_	0.8
W_f_	3	C_L_	4.5
S_L3_	1.8	L	17.02

### Evolution of the proposed antenna design

**Fig. 2:**
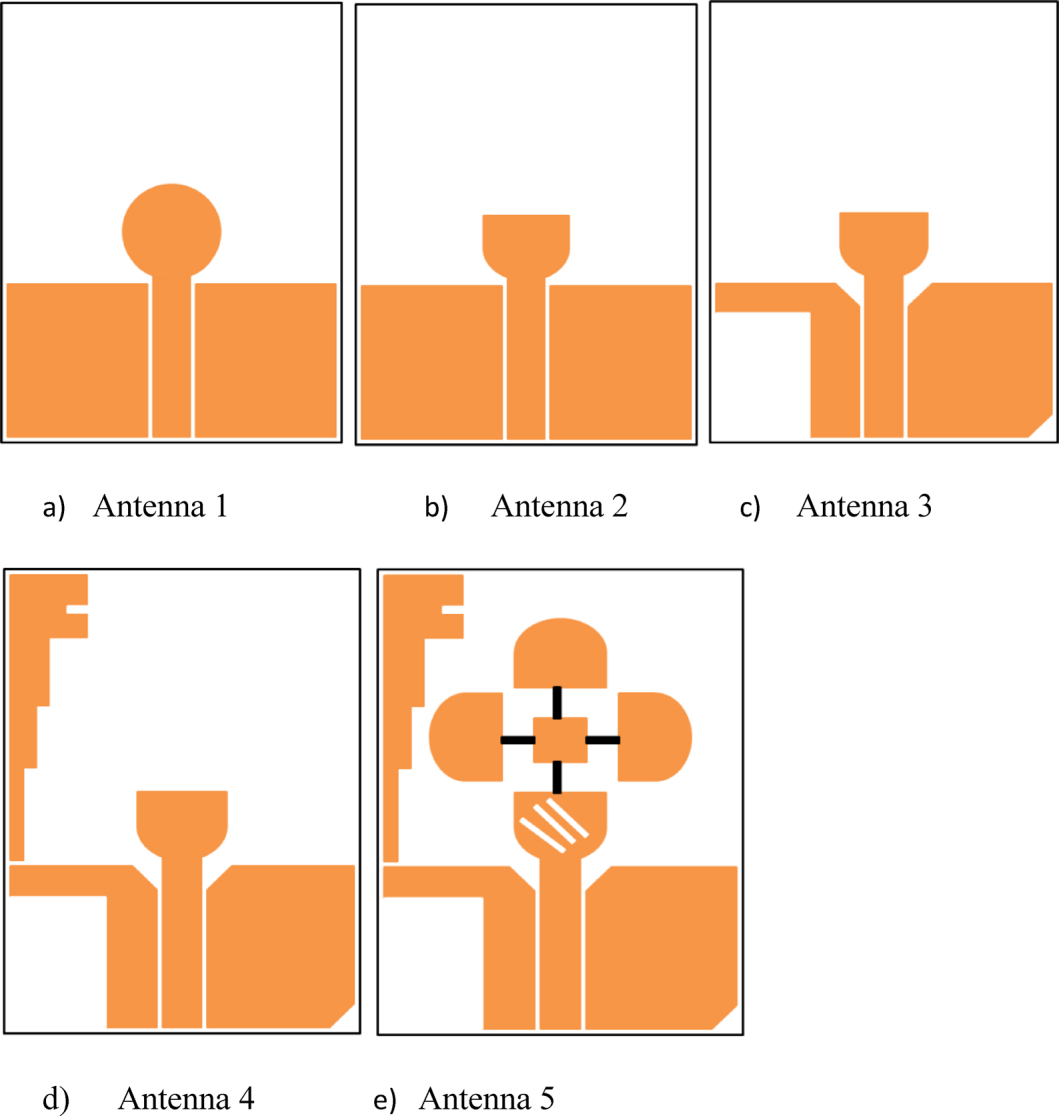
The design flow of the five stages of antennas for evoluation studies (**a**) Antenna 1 (**b**) Antenna 2 (**c**) Antenna 3 (**d**) Antenna 4 (**e**) Antenna 5.

**Fig. 3:**
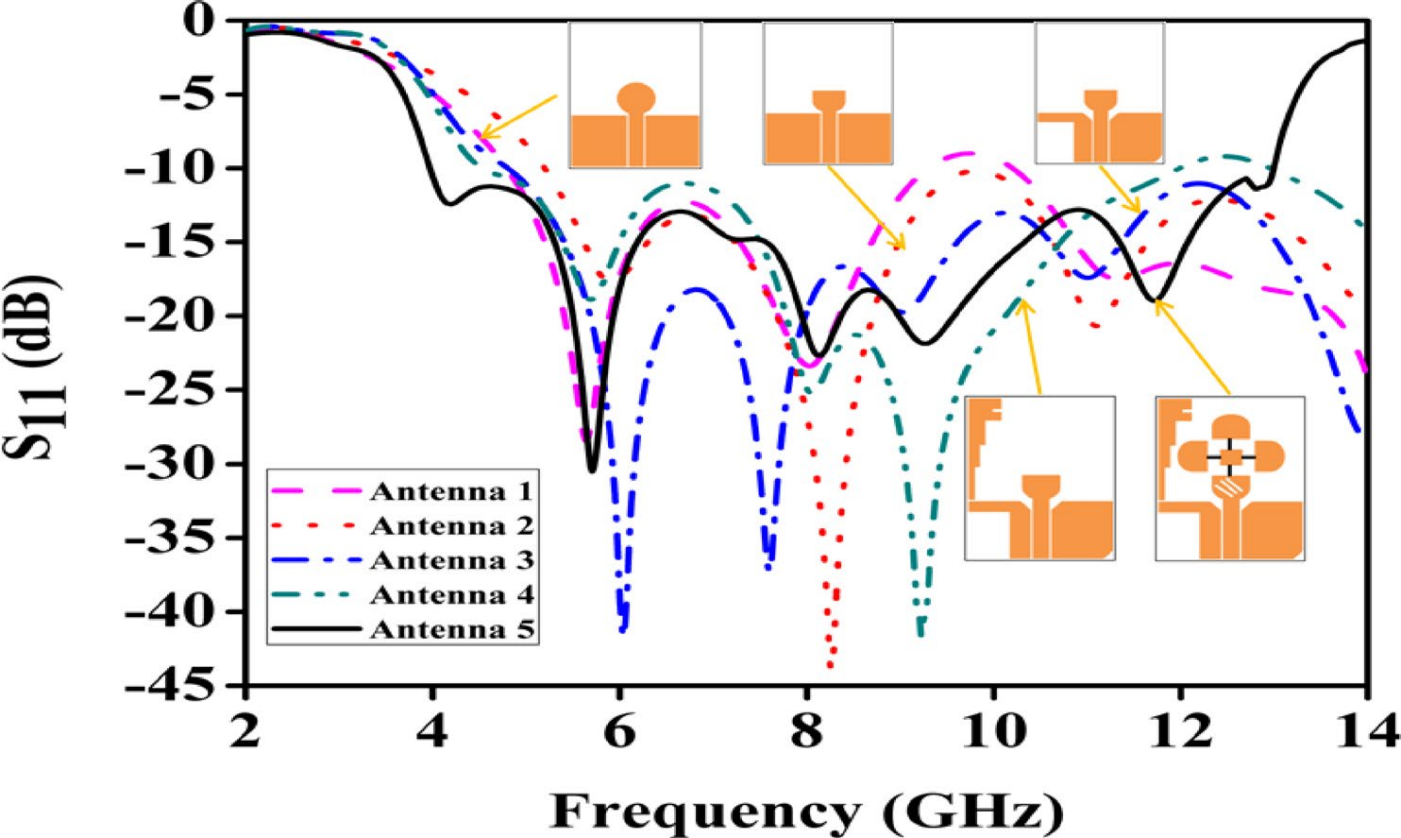
Parametric analysis.

**Fig. 4:**
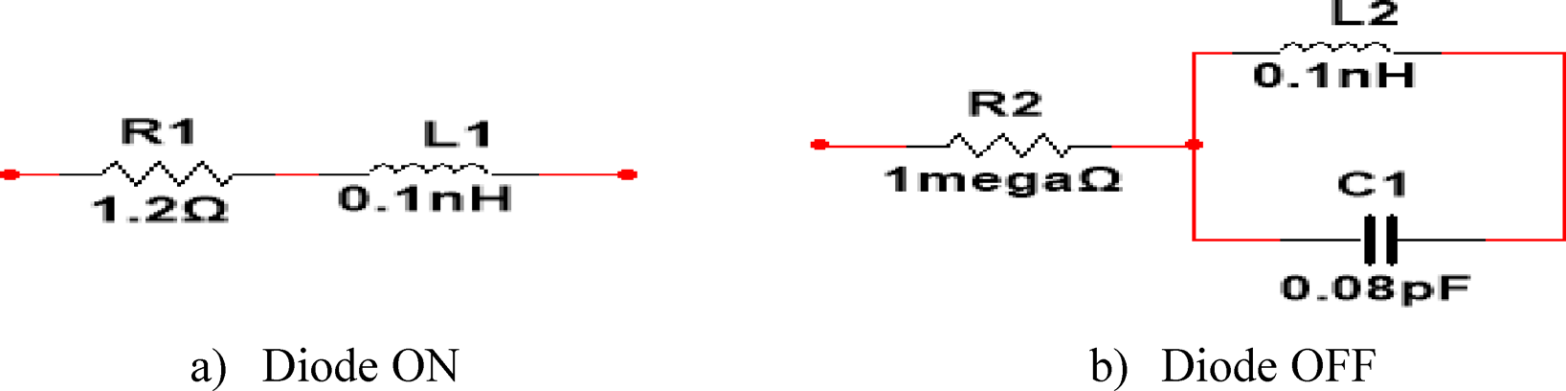
Biasing circuit.

The proposed antenna design progresses through five stages, as shown in Fig. 2a to e. The simulated return loss (S_11_) and axial ratio for these five stages are presented in Figs. 3 and 4. Additionally, the impedance bandwidth and AR bandwidth for the five levels of UWB antennas are compared in Table 2. The design starts with a semicircular UWB antenna with an asymmetric CPW feed, targeting a resonance frequency of (3.89–12.94) GHz, as depicted in Fig. 3. The initial design shows that the UWB and ground plane are etched on one side of the substrate, withthe opposite side left blank. The antenna features a circular radiating patch, and a vertically attached metallic strip extends from the left end of the ground plane, forming a laterally inverted F shape. This F-shaped structure improves impedance matching and bandwidth. The antenna is powered by a 50Ω SMA connector, and the optimal geometry of the presented reconfigurable UWB antenna is illustrated in Fig. 1.

### Antenna design stages

In this analysis, Antenna 1 represents a conventional circular-shaped antenna designed for UWB applications, utilizing a CPW feed line and a ground plane on the front side. The circular patch and CPW feed line are merged, and a 50Ω input impedance is applied. Simulations of this antenna resulted in low resonance at the target frequency. For Antenna 2, slight modifications were made to change the circular shape into a semi-circular one. Simulations showed significant improvement in the initial resonance, but it did not cover the UWB range and had a narrower bandwidth compared to Antenna 1. To address this, Antenna 3 involved removing a portion of the left-side ground plane, leading to improved results in the starting frequency and bandwidth. Finally, Antenna 4 achieved the desired UWB frequency range by adding an inverted F-shaped patch, resulting in proper UWB coverage. Additionally, three semi-circular patches were added to the design and connected via various PIN diodes(BAR64-03WE6327). This allows switching between the semi-circular patches, enabling frequency reconfiguration and improving impedance bandwidth along with gain. This antenna covers S, C, and X bands for wireless communication.

### Switching operation of PIN diodes

. The ON condition in the PIN diode model is realized as a series RL component with small values that acts as a short circuit, allowing the current to pass along the radiator, according to research done on the literature. This phenomenon contrasts with the OFF condition of a PIN diode, which is described as a parallel RLC component with such values and displays an open circuit behavior that prevents current from flowing along the radiator. The switch was implemented as an RLC lumped element model using only resistor values in order to streamline our model by concentrating on the open circuit and short circuit behavior. When the resistor is chosen as a small value of R1 = 1.2Ω and inductance value of L1 = 0.1nH, it acts like a short circuit and permits regular current flow. Conversely, a resistance of 1MΩ and parallel connection of inductance value of L1 = 0.1nH and capacitance C1 = 0.08pF is demonstrated for open circuit behavior, blocking the current route along the radiating structure. The biasing circuits of the diode ON and OFF modes are depicted in Fig. 4.

## Results and discussion

### Simulated and measured results (S_11_)

To evaluate the performance of the UWB antenna, the radiating structure is designed, simulated, and analyzed using CST Microwave Studio. A waveguide port is used as the excitation source for the antenna. It employs a transient solver to assess the parameters such as VSWR, gain, directivity, far-field pattern, return loss (S11).

**Fig. 5:**
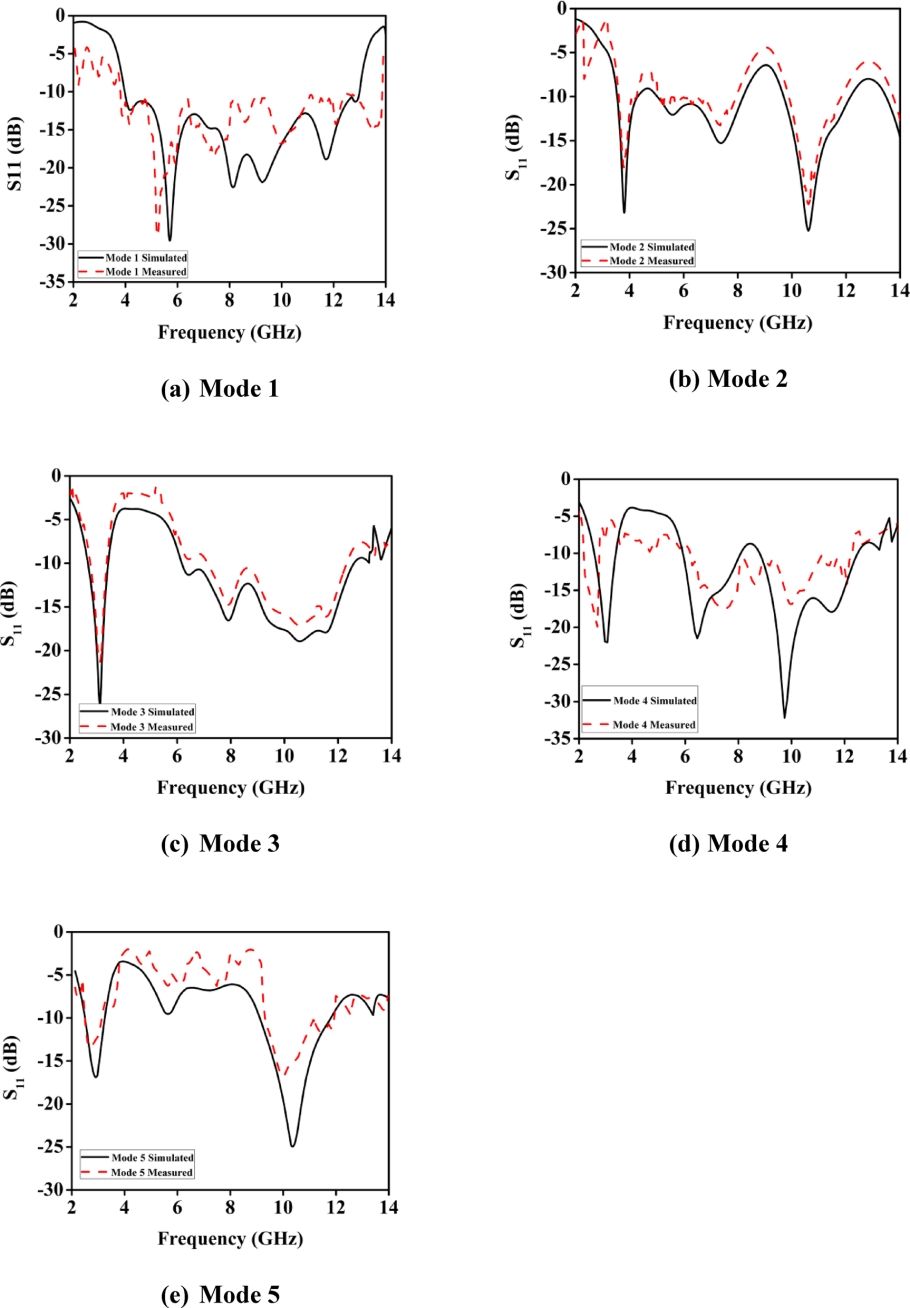
Simulated and observed reflection coefficients for operation modes.

When all Diodes (D1, D2, D3 and D4) are turned OFF, the proposed antenna functions in a UWB mode 1 at (3.89–12.94) GHz with return losses of (30.44dB), respectively. In Mode 2, single diode D1 is turned ON and D2, D3, D4 is turned OFF; the antenna operates in Triple band mode 2, at 3.8, 7.4 and 10.6 GHz, with return losses of 23.3 dB, 23.3 dB, and 25.23 dB respectively . In Mode 3, two diodes D1 and D2 are turned ON and the other two Diodes D3 and D4 are turned OFF, the antenna operates for dual band mode 3 at 3.1 GHz and (6.2–12.5) with a return loss of 26.81 dB and 18.94 dB. In Mode 4, three diodes D1, D2, D3 are turned ON and D4 is turned OFF, the antenna operates in a triple band mode 4 at 3 GHz, 6.4 GHz and 9.7 GHz with a return loss of 22 dB, 21.42 dB and 32.23 dB respectively. In Mode 5 all the diodes are turned ON, the intended antenna works in a dual band mode 5 at 2.7 GHz and (9.12–11.7) GHz, with a return loss of 17.45 dB and 25.32 dB. The proposed inverted F shaped UWB antenna has higher bandwidths (-20 dB return loss) of (3.8-12.94) GHz, 3.8, 7.4, 10.6, 3.1, 3, 6.4,9.7 and (9.12–11.7) GHz. Figure 5 shows the measured and simulated reflection coefficients for the UWB and multiband antenna performances. It is worth noting that the measured results are in good agreement with the simulation results.

**Table 2:** Various switching modes of operation.

Mode	D1	D2	D3	D4	Operation (GHz)	Impedance BW (GHz)	Axial Ratio BW (GHz)	Gain (dBi)	Applications
Mode − 1	OFF	OFF	OFF	OFF	(3.8–12.9)	9.1	(8.2 - 8.4), (10.2–10.9)	6.82	UWB
Mode − 2	ON	OFF	OFF	OFF	3.8, 7.4, 10.6	0.8, 3.2, 2.4	NA	3.19, 4.1, 4.95	Sub 6 GHz 5G, X-band
Mode − 3	ON	ON	OFF	OFF	3.1, (6.2–12.5)	0.8, 4.3	NA	2.2, 5.5	WiMAX, X - band
Mode − 4	ON	ON	ON	OFF	3.0, 6.4, 9.7	0.9, 2.1, 4	NA	3, 2.9, 4.2	WiMAX, Radar
Mode − 5	ON	ON	ON	ON	2.7, (9.12–11.7)	0.8, 2.6	NA	3, 3.8	Wifi, X - band

###  Axial ratio and gain

**Fig. 6:**
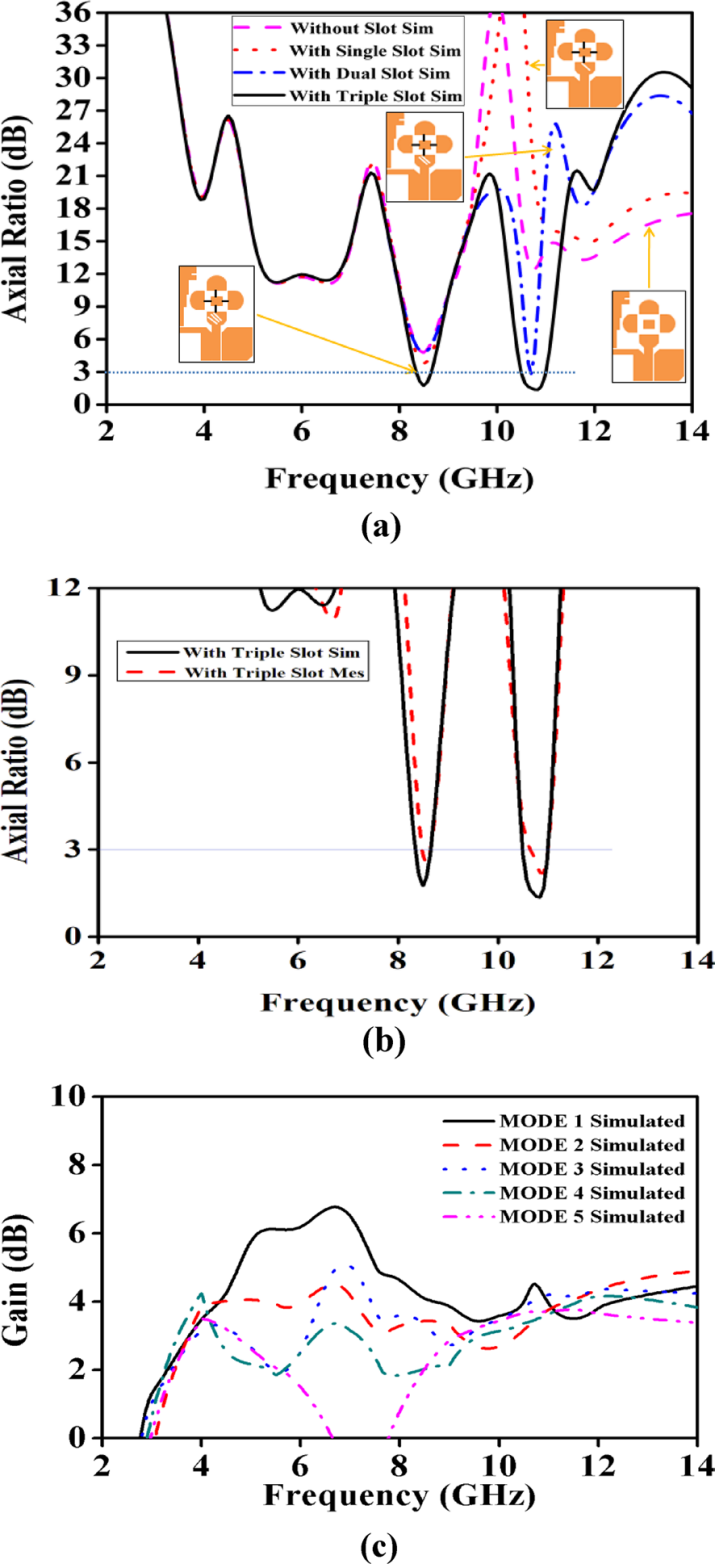
(**a**). Variation of axial ratio with and without the slots (**b**). Comparison of Simulated vs. Measured Axial Ratio (**c**). Simulated Gain of all modes.

Figure 6a- c, shows that when all of the diodes (D1, D2, D3, D4) are turned OFF, the antenna radiates with peak gain of 6.93dBi at (3.82 and 12.94) GHz. By changing the switching states, the antenna is working in a dual band mode with a peak gain of 3 dBi and 3.8 dBi at 2.7 GHz and (9.12–11.7) GHz for all the diodes are turned ON condition. When D1  aloneturned ON, the proposed antenna working in a triple band mode with peak gain values of 3.19, 4.1 and 4.95 dBi at 3.8, 7.4 and 10.6 GHz frequencies. When D1 and D2 are turned ON while D3, D4 is turned OFF, the antenna operates in a Dual resonant mode with a peak gain value of 2.2 and 5.5 dBi at 3.1 and (6.2–12.5) GHz, respectively When D1, D2 and D3 are turned ON while D4 is turned OFF, the antenna operates in a Multi resonant mode with a peak gain value of 3, 2.9 and 4.2 dBi at 3, 6.4 and 9.7 GHz.

**Fig. 7:**
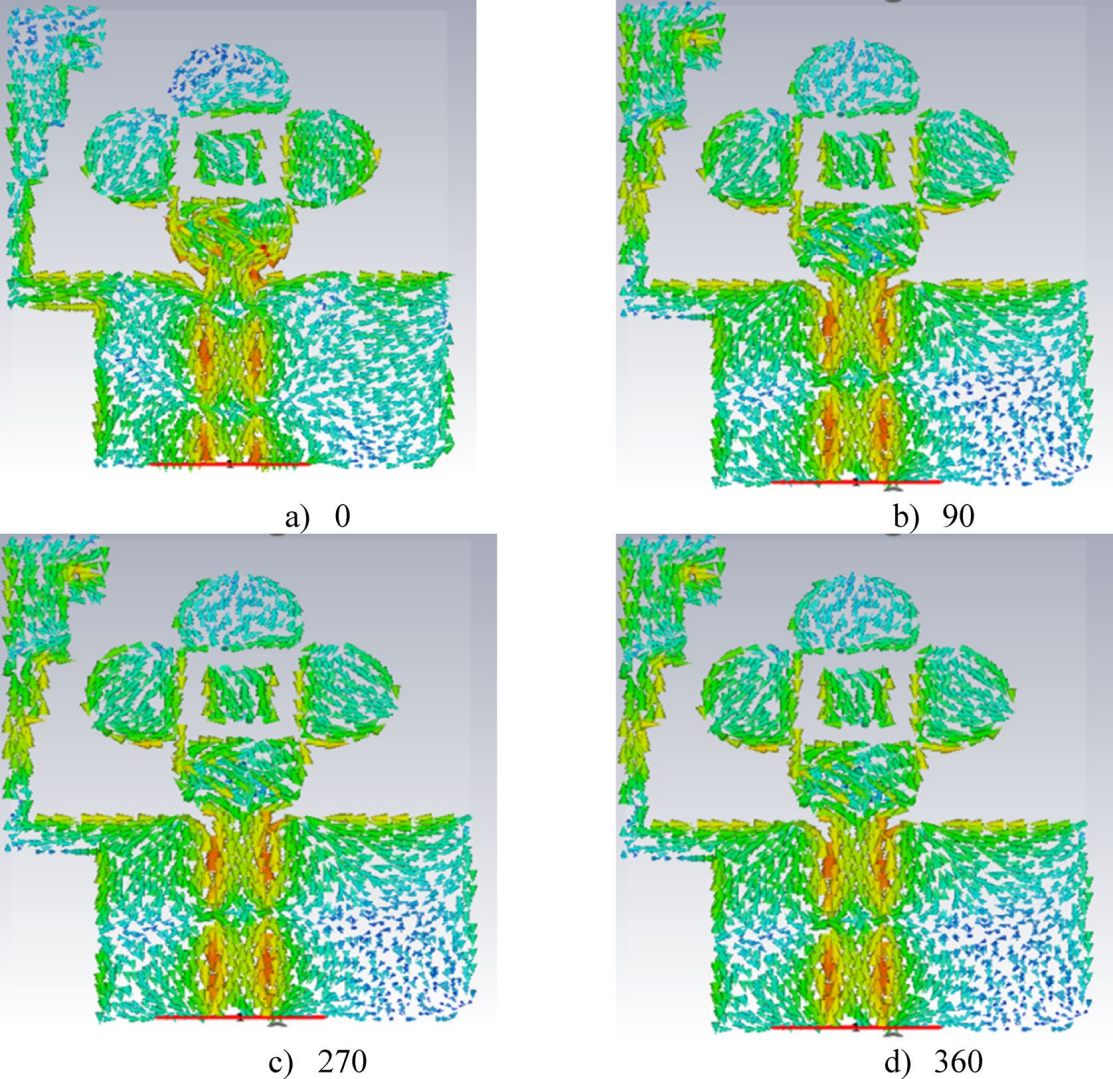
The electric field distributionof the proposed antenna at 10.7 GHz.

This UWB antenna, without any slots, typically operates in linear polarization. To achieve circular polarization, a single slot is introduced in the semi-circular CPW-fed antenna. The design is simulated to obtain axial ratio values above 3 dB. After adding two slots to the same antenna, simulations show values near 3 dB. Finally, a third slot is introduced, and simulations yield the desired axial ratio below 3 dB. The simulated and measured results are shown in Fig. 6b. The placement of the slots around the semi-circular patch generates the necessary phase differences between orthogonal current modes. These slots are typically positioned at specific angles to create a 90° phase shift between the two orthogonal components, essential for circular polarization. The axial ratio of the antenna is influenced by the length and width of the slots. Figure 7 shows the surface current distribution along the radiating circular polarization conducting semi-circular patch at 10.7 GHz, illustrating effective resonance at 0°, 90°, 270°, and 360° for the same frequency.

**Fig. 8:**
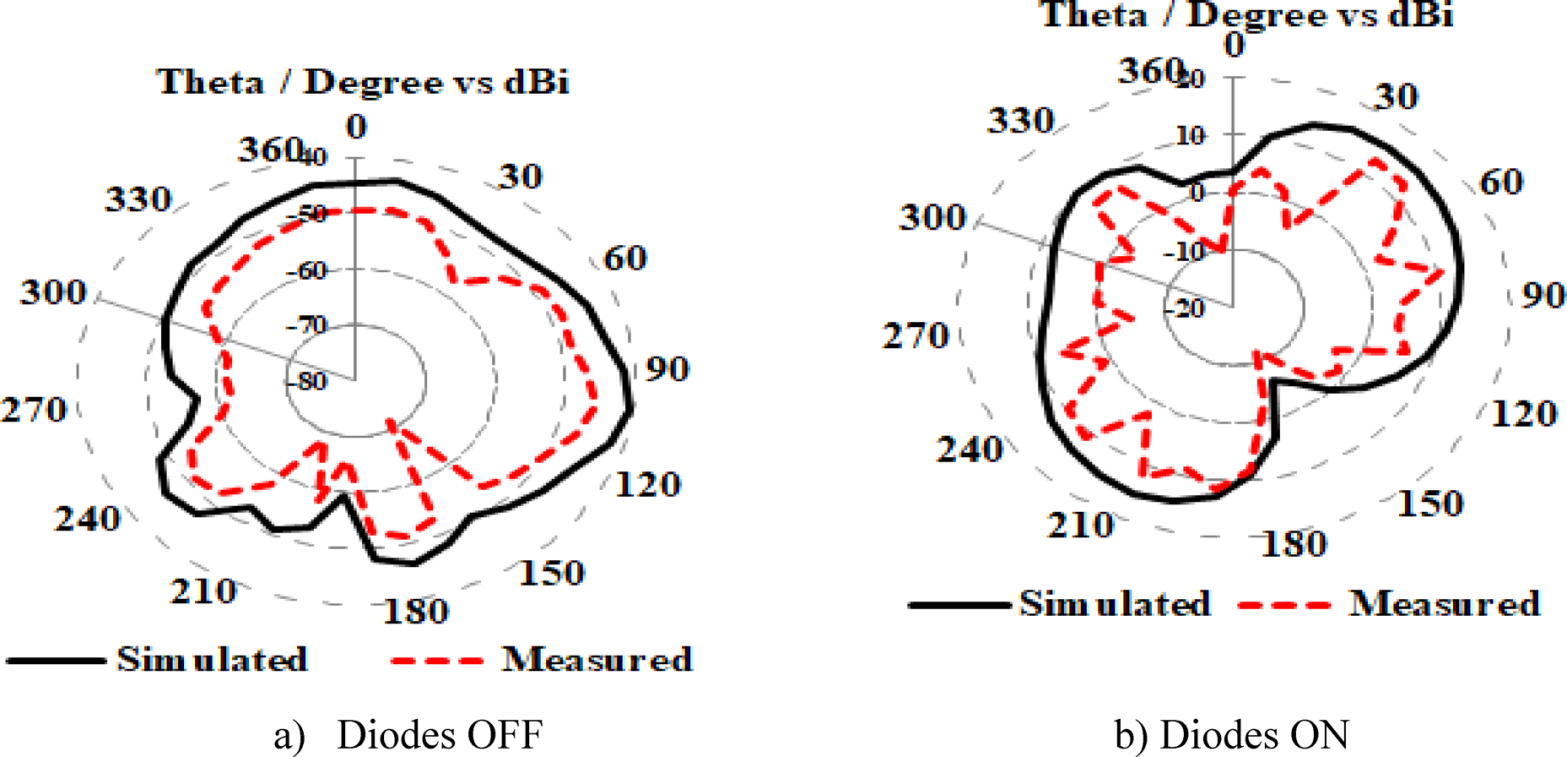
Radiation Pattern of the antenna at 10.7 GHz. (**a**) Diodes OFF (**b**) Diodes ON.

The simulated and measured E-plane radiation pattern at 10.7 GHz frequency band of the proposed antenna exhibits both omnidirectional and bidirectional pattern for all diodes OFF and ON condition, as shown in Fig. 8, whereas the E-plane virtually forms a “figure of eight” shape for low and higher mode two resonant bands. . At 5.7 GHz and 10.7 GHz frequency bands, respectively, the radiation efficiency of the suggested antenna is 90.58 and 92.40%. For the semi-circular shaped UWB antenna in the proposed UWB application the antenna peak gain value of 6.93 dBi at (3.89–12.94) GHz which is sufficient for many wireless applications.

Table 3 summarizes the overall performance of the proposed UWB and multiband communications. The proposed antenna size is reduced with better gain, a maximum number of operating bands, and provides a maximum bandwidth. 

**Fig. 9:**
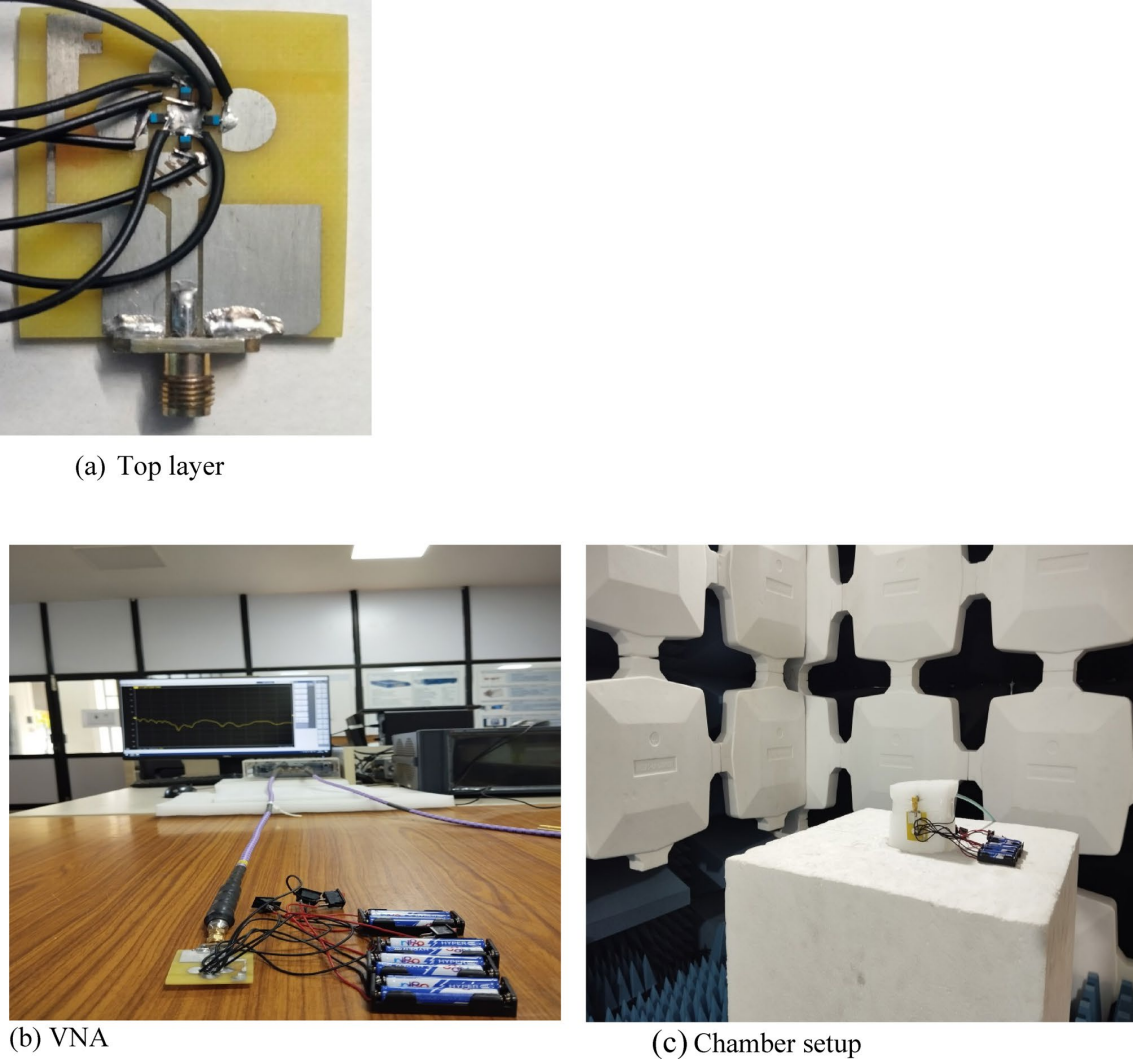
Fabricated antenna and with measurement setup.

As part of the fabrication and testing process, the antenna is tested for its merits such as reflection coefficient and radiation characteristics. Field Fox network analyzer MS2027C VNA Master is used for reflection coefficient measurements. , . The fabricated antenna and the measurement setup is shown in Fig. 9.

**Table 3:** Comparison table of proposed antenna.

Ref.No	Year	Size (mm)	Frequency (GHz)	Gain (dBi)	Application
2	2020	30*30	0.77, 1.43, 2.13, 3.48, 3.84, 5.17, and 6	1.1, 1.3, 1.1, 1.6, 1.7, 1.8, and 2.2	Multiband, WLAN, WiMAX, Sub 6 GHz 5G, and Fixed Satellite Communication
3	2020	55*50	6.02	3.59	NB
5	2023	59.5*35	3.4–5.32	6.02/5.37	WB
7	2017	40*40	(3.1–10.6),3.6 and5.2	5.5 and 6	UWB/C/S
8	2015	80*70	2.78–10.85	4.13–5.83	UWB
9	2018	60*48	(1.7–5.1), 3.2 and 5	NA	UWB/NB
10	2017	45*40	(2.2–11), 2.4 and 5.8	3.2/2	UWB and WLAN
11	2018	60*54	(3.47– 11.07),	1.26,3.58	UWB
13	2021	60*58	3.4–10.2	1.46	UWB
15	2015	40*40	2.5–12	4.2	UWB
22	2024	40*30	(3.28– 17.8),	4.93	UWB, Ku-Band
23	2024	15*18	(4.11–29.77),	1.7–4.82	UWB, Ku, Ka-Band
This work	2024	40*30	(3.9–12.4), 3.8,7.4,10.6, (6.2–12.5), 6.4,9.7, 2.7, (9.12–11.7)	6.93, 3.19,4.1,4.95, 5.5, 2.9,4.2, 3, 3.8	UWB, S,C, X

## Conclusion

A hybrid mode semi-circular shaped antenna has been designed and experimentally validated for UWB and multiband applications. This frequency of the antenna is reconfigured to operate in UWB, triple-band, and dual band depending on its switching condition. When all diodes are turned OFF, the antenna functions in UWB mode (3.89–12.94 GHz). When all diodes are turned ON, it operates in a dual band, covering 2.7 GHz (Wi-Fi) and 9.12–11.7 GHz (X-band). When diode D1 is ON and diodes D2, D3, and D4 are OFF, the antenna functions are in triple-band, covering 3.8 GHz (Wi-Fi), 7.4 GHz (WLAN), and 10.6 GHz (X-band). When diodes D1 and D2 are ON while D3 and D4 are OFF, the antenna operates in dual-band at 3.1 GHz (WiMAX) and 6.2–12.5 GHz (X-band). Finally, when diodes D1, D2, and D3 are ON, and D4 is OFF, the antenna operates in triple-band, covering 3 GHz (WiMAX), 6.4 GHz (satellite communication), and 9.7 GHz (radar communication). The proposed hybrid semi-circular shaped antenna exhibits high efficiency above 90% and average gain above of 5dBi across different modes.

## Data Availability

The datasets used and/or analysed during the current study available from the corresponding author on reasonable request.
